# Recyclable Printed Liquid Metal Composite for Underwater Stretchable Electronics

**DOI:** 10.1002/smsc.202400553

**Published:** 2025-03-31

**Authors:** Chi‐hyeong Kim, Jinsil Kim, Jiaxin Fan, Meijing Wang, Fabio Cicoira

**Affiliations:** ^1^ Department of Chemical Engineering Polytechnique Montreal Montréal H3T 1J4 Canada

**Keywords:** conducting composites, liquid metals, printed electronics, recycling, stretchable electronics, sustainable electronics

## Abstract

Multifunctional stretchable conductors are crucial components in fully stretchable circuits for wearable bioelectronics. Conductive composites made from liquid metal (LM) fillers and polymer matrices have garnered significant interest due to their high electrical conductivity, adjustable mechanical properties, biocompatibility, and recyclability. Herein, a printable LM composite is developed using a custom‐designed block copolymer to ensure electromechanical stability in both wet and dry conditions. The LM composite demonstrates high conductivity (around 10^5^ S m^−^^1^), stretchability up to 500%, and maintains stable resistance with cyclic strain ranging from 0 to 50% for over 16 h, in both ambient and aqueous environments. Furthermore, bulk LM is successfully recovered from printed composites using green solvents, supporting the composite's recyclability.

## Graphical abstract

This work presents a multifunctional liquid metal (LM) microdroplet‐based composite with a custom‐designed block copolymer as the matrix, optimized for printing. The composite exhibits high conductivity, stretchability, durability in dry and aqueous environments, and recyclability of LM using green solvents. These properties highlight its potential for sustainable wearable electronics under aqueous environments.

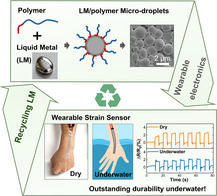

## 1 Introduction

Soft wearable electronics are in high demand for various applications, including electronic skin and wearable healthcare monitoring systems.^[^ ^1^, ^2^, ^3^, ^4^, ^5^, ^6^ ^]^ For these applications, devices based on stretchable conductors are desirable due to their ability to conform closely to the surface of biological tissues.^[^ ^7^, ^8^ ^]^ To ensure the stability and durability of wearable electronic devices, these conductors must retain consistent conductivity when subjected to repeated deformation and exposure to aqueous environments, such as rainwater or sweat.

Significant effort has been dedicated to developing multifunctional stretchable conductors to meet the key requirements of wearable electronics. Conducting polymers, often mixed with additives to enhance electrical conductivity (0.1–1000 S cm^−1^),^[^ ^9^, ^10^ ^]^ stretchability,^[^ ^11^ ^]^ recyclability,^[^ ^12^ ^]^ and self‐healing properties,^[^ ^13^ ^]^ have been employed as active components in stretchable devices, such as sensing electrodes and transistor channels. Additionally, they have been explored for interfaces with biological tissues, exhibiting biocompatibility and electrical stability in aqueous environments.^[^ ^14^, ^15^ ^]^ Conducting composites, which incorporate electrically conductive fillers (e.g., metal nanoparticles^[^ ^8^, ^16^ ^]^ and carbon‐based materials^[^ ^12^ ^]^) within a polymer matrix, have been investigated as electrodes and interconnects in flexible and stretchable electronics, achieving high electrical conductivity (1000–70 000 S cm^−1^).^[^ ^10^ ^]^ However, these composites may exhibit electrical instability under repeated mechanical deformation.^[^ ^17^, ^18^ ^]^ The electromechanical properties of stretchable conductors can be improved using fillers based on gallium‐based liquid metals (LMs), such as eutectic gallium indium (EGaIn) and gallium indium tin alloy (galistan). These materials have low melting points (below room temperature), high conductivity (≈10^6^ S m^−1^), and are biocompatible.^[^ ^19^ ^]^ Given that gallium and indium are critical materials, considerable attention has been directed toward their recycling.^[^ ^5^, ^20^, ^21^, ^22^ ^]^

LM composites are typically formed by breaking down bulk LM into micro‐ or nanodroplets, which are dispersed in a fluid, such as uncured polydimethylsiloxane (PDMS) or a liquid polymer solution, through vigorous rotary (shear) mixing^[^ ^23^ ^]^ or sonication.^[^ ^24^, ^25^ ^]^ These suspensions can be used as inks for various patterning techniques, such as stencil printing,^[^ ^26^ ^]^ spray coating,^[^ ^27^ ^]^ and direct ink writing.^[^ ^24^ ^]^ However, immediately after processing, the composites exhibit insulating behavior or low conductivity due to the presence of the insulating shell of gallium oxide (Ga_2_O_3_) and the polymer matrix.^[^ ^28^ ^]^ Activation processes, such as mechanical compression or extension,^[^ ^22^, ^29^, ^30^, ^31^, ^32^ ^]^ laser sintering,^[^ ^27^ ^]^ or acid treatment,^[^ ^24^ ^]^ are typically used to break the oxide layer and the polymer matrix, forming electrically conductive pathways within LM composites.^[^ ^33^ ^]^

Silicone‐based elastomers such as PDMS and Ecoflex are commonly used as matrices for LM composites in wearable electronics due to their high stretchability, chemical inertness, and biocompatibility.^[^ ^34^, ^35^, ^36^ ^]^ Recently, a silicone‐based water‐resistant LM composite with long‐term stability underwater was reported.^[^ ^37^ ^]^ However, the limited solubility of PDMS complicates LM recycling. To improve the dispersion in polymer solutions, the LM droplets can be functionalized with polymer ligands through interactions between surface oxides and functional groups, such as hydroxyls.^[^ ^25^, ^38^, ^39^ ^]^ High‐resolution printing (≈50 μm linewidth) of poly(styrene sulfonate)‐grafted LM composites using meniscus‐guided direct writing has yielded materials with high conductivity (1.5 × 10^6^ S m^−1^) and stable electromechanical properties, with resistance increasing by only 8 at 200% strain and 40 at 500% strain.^[^ ^24^ ^]^ A composite of poly(vinyl alcohol) (PVA) and LM has been used to develop a highly conductive (1.3 × 10^5^ S m^−1^) 3D printing ink compatible with various substrates,^[^ ^29^ ^]^ which has enabled applications in alarm systems, object locators, and highly sensitive wearable pressure and motion sensors. The LM could be recycled through alkaline treatment and reused for ink preparation. While LM composites with excellent electromechanical performance, improved sustainability, and good printability have been developed, further research is needed to achieve multifunctional LM composites that are compatible with various substrates and deposition techniques, offering electrical and mechanical stability, water resistance, and end‐of‐life material recovery.

In this study, we report a printable LM composite based on EGaIn and a custom‐designed block copolymer, poly(acrylic acid)‐*b*‐poly(styrene‐*ran*‐butyl acrylate) (PASTA). The combination of PASTA with LM provided suspension stability and enabled the printing on stretchable thermoplastic polyurethane (TPU). After activation, achieved through acetic acid exposure and mechanical stretching, the PASTA‐grafted EGaIn (LM/PASTA) composite exhibited high conductivity (in the 10^5^ S m^−1^ range), high stretchability (500%), and stable electromechanical performance with minimal variations under cyclic strain in dry and wet conditions. The successful demonstration of a wearable strain sensor with nearly identical performance in air and underwater highlights the potential of our LM/PASTA composite for practical applications. Furthermore, we successfully recycled LM from the printed LM/PASTA electrodes using green solvents. Our recyclable and electromechanically durable materials offer promising contributions to the advancement of emerging soft wearable electronics.

## 2 Result and Discussion

### 2.1 Synthesis of a Custom‐Designed PASTA

**Figure 1:**
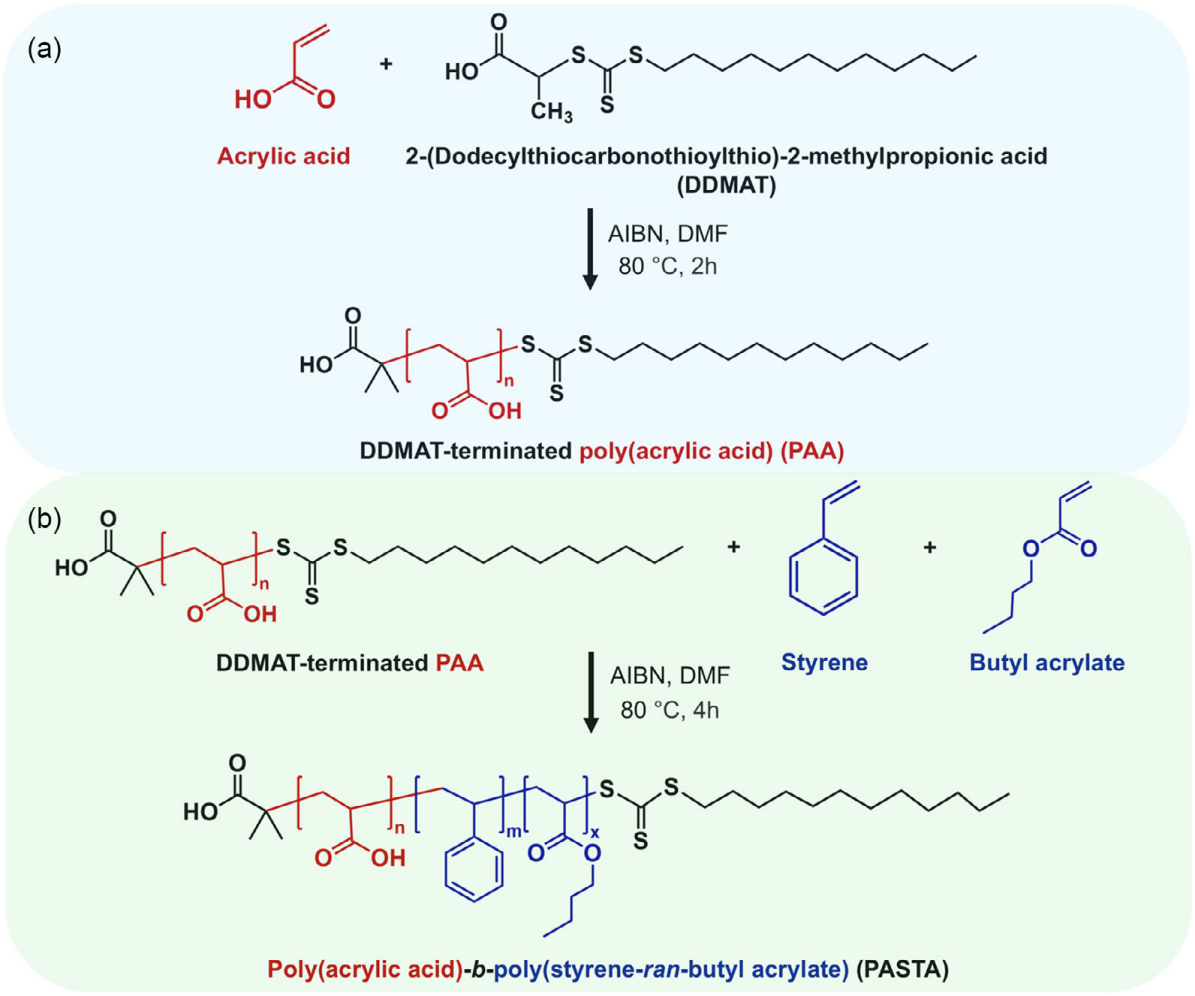
Scheme of polymer synthesis. a) DDMAT‐terminated PAA was synthesized by RAFT, and b) PASTA was synthesized by random polymerization from a DDMAT‐terminated PAA chain (refer to Section 4 for details).

We designed and synthesized a novel block copolymer, PASTA (**Figure** **1**), to develop a multifunctional LM composite with high electrical conductivity and electromechanical stability, suitable for direct ink printing (refer to Section 4 for details).

Anchoring groups for metal oxides are typically hydrophilic (e.g., carboxylate or alcohol).^[^ ^38^ ^]^ Consequently, LM composites based on homopolymers containing such groups, like poly(acrylic acid) (PAA) or PVA, tend to be hydrophilic and, in some cases, water‐soluble. To prevent dissolution and enhance water resistance, we designed a block heteropolymer in which the anchoring carboxylic groups are attached to a minor PAA block, while the major poly(styrene‐ran‐butyl acrylate) block is hydrophobic.

The synthesis was carried out through reversible addition–fragmentation chain transfer (RAFT) polymerization, followed by random free‐radical polymerization. In the first step, using a chain transfer agent (DDMAT), we synthesized DDMAT‐terminated PAA (Figure 1a), which serves as an anchoring block to the surface of the LM droplets by bonding carboxyl groups to the native gallium oxide,^[^ ^38^ ^]^ enabling the formation of a stable LM suspension suitable for printing. Next, a poly (styrene‐ran‐butyl acrylate) block was grown from DDMAT‐terminated PAA (Figure 1b). Butyl acrylate was chosen to impart softness and elasticity to the polymer, due to the relatively high mobility of its butyl ester side chain,^[^ ^40^ ^]^ while styrene was incorporated to enhance mechanical strength, owing to its aromatic ring structure.^[^ ^41^ ^]^ Preliminary tests indicated that an optimized ratio of monomers in the poly(styrene‐ran‐butyl acrylate) block yielded a polymer with both stretchability and strength. ^1^H‐NMR and gel permeation chromatography (GPC) confirmed the successful synthesis of PASTA through well‐controlled polymerization (Figure S1 and S2, Supporting Information). Mechanical characterization (Figure S3, Supporting Information) via stress–strain testing and differential scanning calorimetry (DSC) (Figure S4, Supporting Information) demonstrated the high stretchability of PASTA (≈1000%).

### 2.2 LM Ink Preparation and Printing LM/PASTA

**Figure 2:**
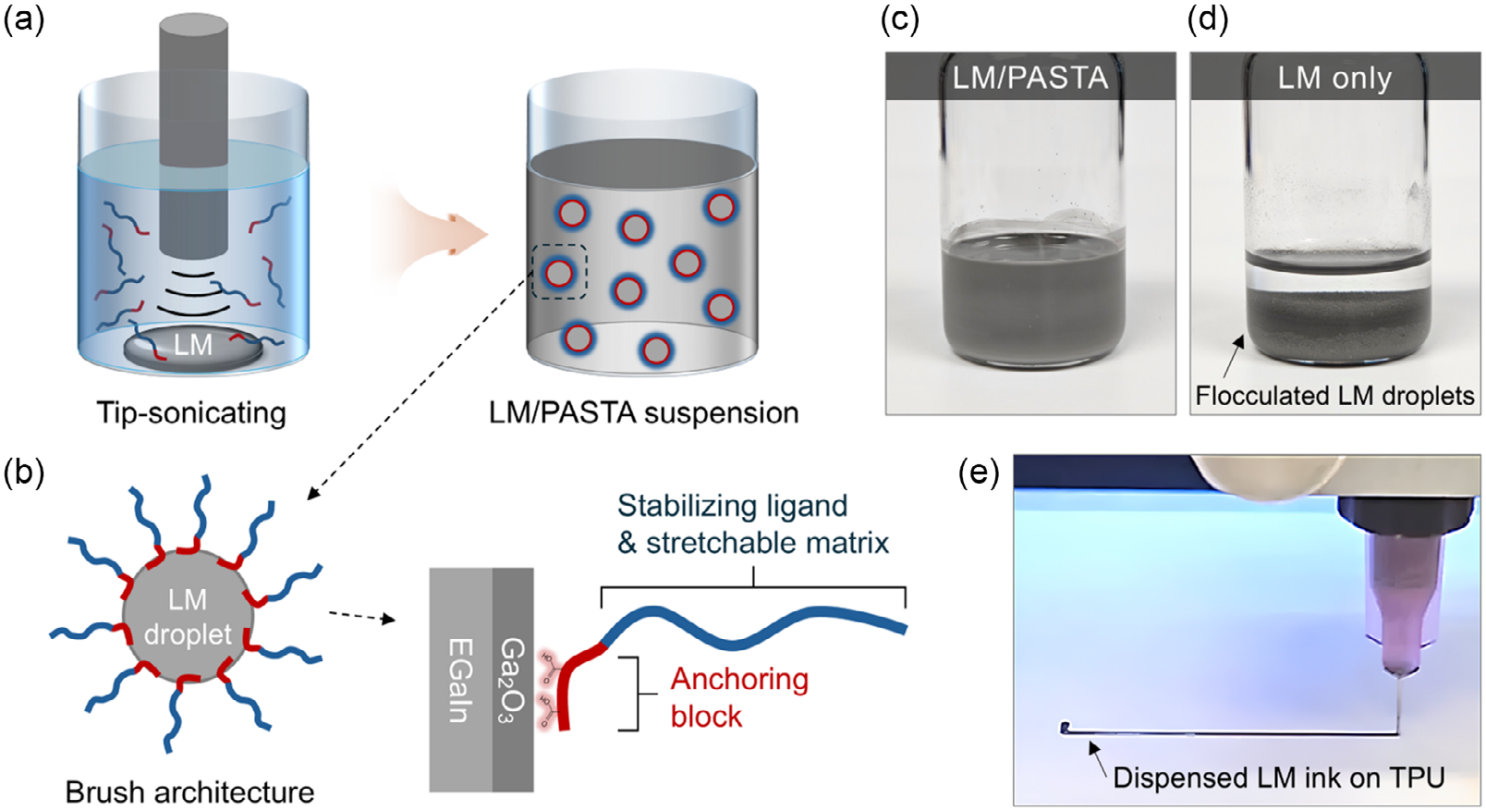
a) Schematics of a LM/PASTA suspension prepared by tip sonication in toluene and b) a PASTA‐grafted LM droplet with polymer brush architecture. Digital photograph of c) LM/PASTA ink and d) control LM ink without the polymer. e) Digital photograph of the printed LM/PASTA ink on TPU.

To prepare the LM suspension ink, EGaIn was added to a PASTA solution in toluene and broken down into microscale droplets via tip sonication (**Figure** **2**a). Composites were prepared using different LM‐to‐PASTA ratios, with the materials produced with 15, 35, and 70 mg PASTA in the solution designated as P15, P35, and P70, respectively (refer to Section 4 for details). The dissolved PASTA grafted onto the droplets via PAA blocks, forming a polymer brush architecture (Figure 2b) that led to the formation of a stable suspension.^[^ ^42^, ^43^ ^]^ The stability of the LM/PASTA suspension was maintained over time (Figure 2c,d and Figure S5, Supporting Information), making it suitable for printing.^[^ ^24^ ^]^

### 2.3 Microstructure and Activation of LM/PASTA

**Figure 3:**
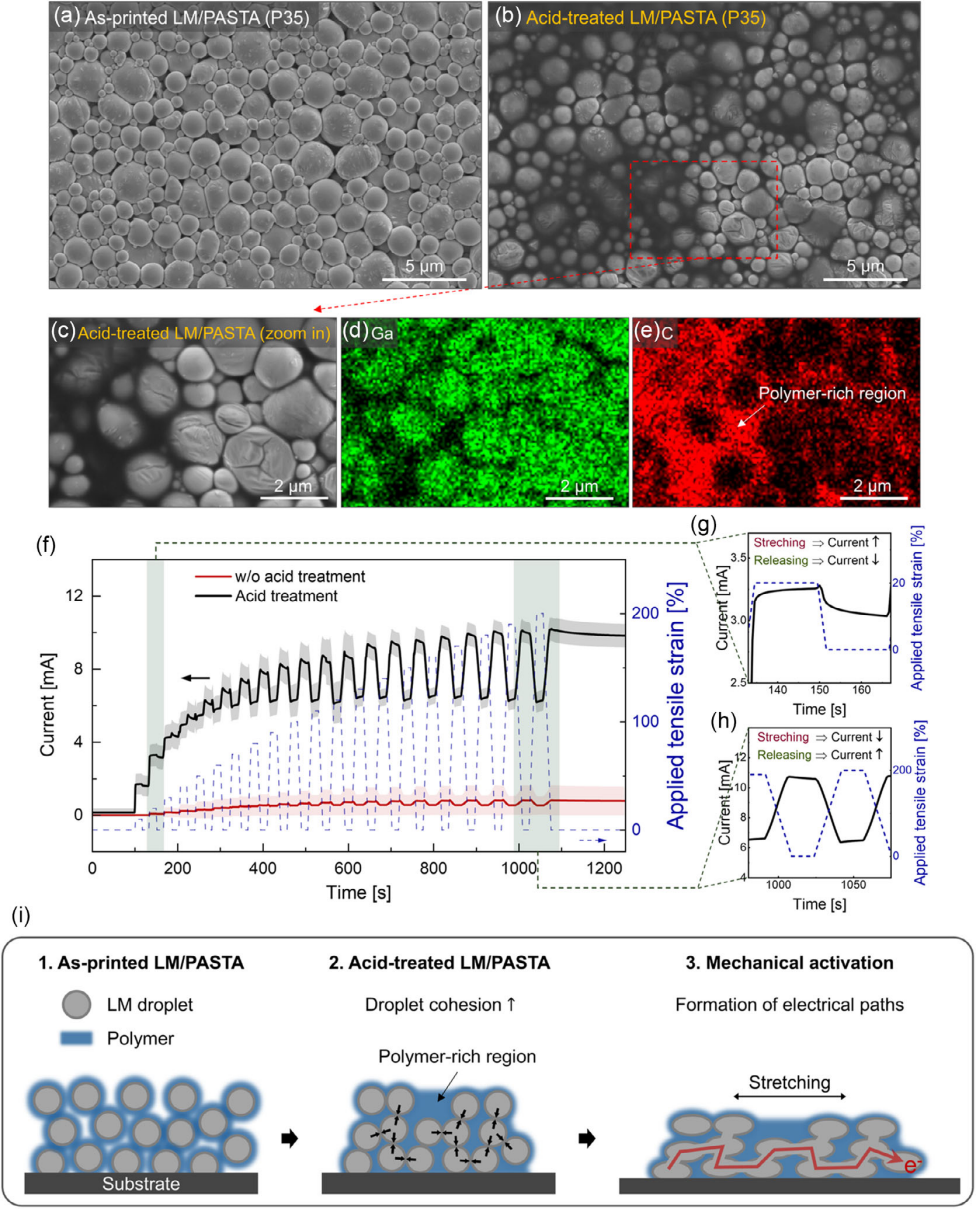
SEM images of a) as‐printed and b) acid‐treated LM/PASTA composites (P35) on TPU. c) Magnified SEM image to highlight the EDX mapping of d) Ga (green) and e) C (red). f) Average current versus time plot showing the currents of the LM (P35) composites during mechanical activation alone (red curve) and combined chemical and mechanical activation (black curve). A constant voltage of 30 mV was applied. The shaded area around the curves represents the standard deviation extracted from three (mechanical treatment) and five (combined treatment) samples. The dashed blue line (right axis) represents the applied strain, which was increased in 10% increments from 0 to 200% (right axis). Magnified plots of current change at g) 20% strain and h) 200% strain in the stepwise mechanical activation process. i) Scheme of the acid treatment and mechanical activation processes of the LM composite.

Printing on a TPU substrate (Figure 2e; see Section 4 for details) resulted in the formation of an LM composite (P35, thickness ≈14.4 μm, Figure S6, refer to Supporting Information for details) consisting of nearly circular droplets with an average size of ≈1.2 μm (**Figure** **3**a). Initially, the composites exhibited insulating behavior because of the presence of the gallium oxide layer and the polymer matrix surrounding the droplets, making activation necessary to enhance electrical conductivity.^[^ ^44^ ^]^ To gain insight into the activation process, we first investigated the effect of chemical and mechanical treatment individually, followed by a combined approach. Chemical treatment was performed by exposing the composite to a solution of 10 vol% acetic acid in acetone. Acetone was chosen as the solvent because it partially dissolves PASTA before evaporation, facilitating the penetration of the acid through the composite. Scanning electron microscopy (SEM) images reveal that after chemical treatment the spacing between the LM droplets increased (Figure 3b and Figure S7, Supporting Information). The energy‐dispersive X‐ray spectroscopy (EDX) maps of gallium and carbon (Figure 3c–e) strikingly reveal a significantly higher carbon content in the gaps between the LM droplets, highlighting these areas as polymer‐rich regions.

This suggests that acetic acid may partially remove the surface gallium oxide, to which PASTA is anchored, causing the polymer to detach and occupy the space between the droplets, leading to morphological changes. This acid treatment also led to optical color alterations in the composite (Figure S8, Supporting Information), a phenomenon similar to the one previously reported for DMSO‐treated LM composites.^[^ ^45^ ^]^ The chemical reactions involved in this process are explained in a later section.

However, following this treatment, the composites remained insulating, indicating that exposure to acetic acid solution alone was insufficient to establish electrical pathways between the LM droplets. To increase the conductivity, we investigated the effect of mechanical treatment (red curve, Figure 3f) by applying stepwise uniaxial tensile strain (0–200%) and then combined this with chemical treatment (black curve, Figure 3f). The composite activated by stretching alone (red curve) exhibited a current increase from the noise level (10^−8^–10^−7^ mA, Figure S9, Supporting Information) to ≈1 mA after activation at 10–50% strain, with poor sample‐to‐sample reproducibility (Figure S9, Supporting Information). The average conductivity of the activated samples was ≈2.0 × 10^4^ S m^−1^.

The combined treatment, consisting of acetic acid treatment followed by mechanical activation, was more effective to increase electrical conductivity (Figure 3f, black curve).

The highest current observed in the activated samples was in the 10 mA range, corresponding to an average conductivity of ≈3 × 10^5^ S m^−1^, which is one order of magnitude higher than that obtained by mechanical activation alone. The current showed a steep increase upon applying strains in the 10–50% range, followed by a gradual increase up to 200% strain, indicating that the LM composite was nearly fully activated at ≈50% strain. Treatment with acetone followed by mechanical activation resulted in a conductivity similar to that obtained after mechanical activation alone (Figure S11, Supporting Information), confirming that acetic acid is responsible for chemical activation.

Interestingly, different electromechanical coupling behaviors were observed across various activation strain ranges. In the 10–30% strain range (Figure 3g), the composites exhibited a higher current when stretched than when released, even under repeated 20% strain for 1000 cycles (Figure S10, Supporting Information), as already observed for a few other LM composites.^[^ ^46^ ^]^ For applied strains beyond 40%, the current in the stretched state was lower than in the released state (Figure 3h), a behavior commonly observed in stretchable conducting composites.^[^ ^8^, ^12^, ^24^, ^26^ ^]^ Activation by acid treated and 50% strain was performed to achieve a common electromechanical coupling behavior (current in the stretched state lower than in the released state).

Based on the above results, we illustrated the activation process with a schematic (Figure 3i). Initially, the composites are insulating due to the gallium oxide and polymer layer surrounding the droplets. Upon acid treatment, the grafted polymer is removed from the droplets and diffuses toward the surface of the composite, forming a polymer‐rich region. This migration of polymer chains likely reduces the thickness of the insulating barrier around the LM droplets and enhances droplet cohesion. Finally, tensile activation physically ruptures the insulating gallium oxide, allowing LM leakage that forms conductive pathways within the polymer matrix. Acid treatment likely facilitates the formation of these conductive paths during mechanical activation. Thus, the combination of acid treatment and mechanical strain successfully activated the LM/PASTA, resulting in high conductivity.

To optimize the polymer content, we compared materials prepared with different PASTA contents (P15, P35, and P70, Table S1 and Figure S12, Supporting Information). P15 exhibited conductivity similar to that of P35 but with poor reproducibility and nonuniform droplet sizes. In contrast, P70 exhibited a conductivity approximately one order of magnitude lower, likely due to an excess of insulating polymer between the LM droplets. Consequently, P35 composites were used for the remainder of this study, as they provided high conductivity and a uniform morphology.

### 2.4 Electromechanical Characteristics in Dry and Wet States

**Figure 4:**
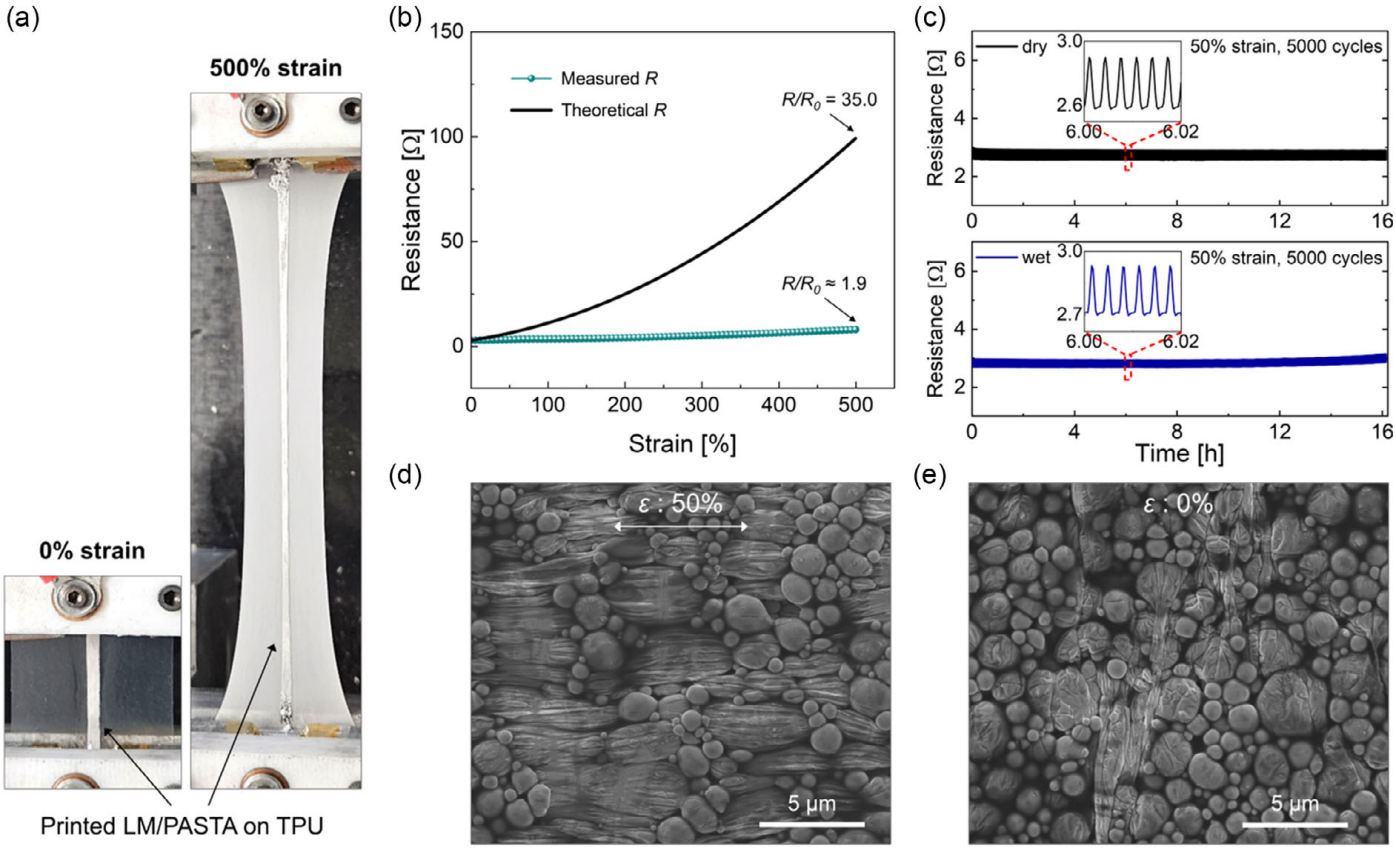
a) Digital photograph of a LM/PASTA composite (2 × 25 mm^2^) printed on thermoplastic polyurethane (TPU) at 0% and 500% strains. b) Measured and theoretical resistances versus strain of the printed LM composite. The sample was stretched at a speed of 2 mm s^−1^. c) Resistance versus time of LM/PASTA (P35) over 5000 cycles of 50% strain conducted over 16 h under dry and wet conditions. The insets show the magnified plots (6.00–6.02 h). For the wet conditions test, the LM composite was wet with water supplied by a syringe pump (Figure S13, Supporting Information). SEM images of d) a uniaxially stretched (50%) sample and e) a released (0%) sample used in the durability test.

To further investigate the electromechanical properties, we conducted electromechanical characterization of the composites activated by stretching them 20 times to 50% tensile strain. The composites were subjected to a continuous tensile strain of up to 500% (**Figure** **4**a), and the change in the resistance was monitored (Figure 4b). The samples with an initial resistance of ≈3 Ω at 0% strain exhibited a resistance increase of ≈5% at 30% strain, ≈20% at 100% strain, and ≈40% at 200% strain. The change in the measured resistance is much smaller than that of the theoretical value, which can be expressed with the equation (see Note, Supporting Information for details) below.^[^ ^26^ ^]^

(1) $$R\left(t\right)=R_{0}{\left(1+\varepsilon \left(t\right)\right)}^{2}$$

where R(t) is the theoretical resistance at a given time (*t*), which was derived from a typical resistance equation for materials with constant conductivity and volume. $R_{0}$ is the resistance of the unstretched LM composite, and ε(t) is the applied strain at the time *t*. Based on Equation (1), the theoretical resistance of LM/PASTA is expected to increase to ≈100 Ω (3500%) at 500% strain; however, a resistance increase of only ≈8 Ω (≈185%) was observed (Figure 4b).

The electromechanical durability test was performed by applying repeated tensile strain (loading–unloading) while monitoring the resistance change (Figure 4c), under both dry and wet conditions. Under both conditions, the composites showed stable performance over 5000 cycles of 50% strain conducted over 16 h (additional results are available in Figure S14, Supporting Information) and remained stable even after the same number of cycles of 100% strain (Figure S15, Supporting Information). The stretchable PASTA matrix effectively prevented significant cracking and delamination of the composite from the substrate during the mechanical deformation. Moreover, the stability under wet conditions can be attributed to the hydrophobic blocks of PASTA, which protect the polymer matrix from swelling and weakening in water.

To gain insight into the electromechanical behavior of the composite, we examined the morphology of a stretched (50% strain) and a released sample after the durability test (5000 cycles). Overall, SEM images showed that the durability test did not induce major morphological changes, such as cracks or droplet ruptures, which align with the observed electromechanical stability. Upon stretching (Figure 4d), relatively large droplets on the surface of the composite were elongated in the direction of the applied tensile strain while small droplets remained spherical shapes. In the released state (Figure 4e), most large droplets returned to nearly circular shapes and showed wrinkles due to stretching.

Overall, the materials reported here exhibited stretchability and electrical performance comparable to previously reported LM composites (Table S2, Supporting Information), while demonstrating exceptional electromechanical durability in water, an area still largely unexplored. These characteristics make these composites ideal materials for electrodes and interconnects in wearable electronics, especially when exposed to aqueous environments such as sweat or rain.

### 2.5 Strain Sensor Based on Printed LM Composite

**Figure 5:**
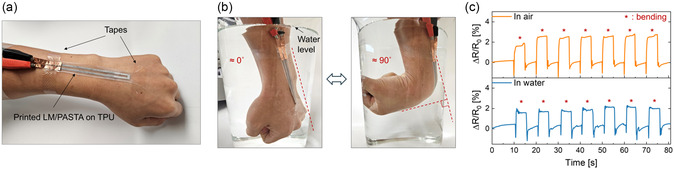
a) Digital photograph of printed LM composite‐based sensor on TPU, fixed on the wrist of a volunteer using Tegaderm tape. b) Wrist with strain sensor repeatedly bent around underwater. c) Resistance change versus time upon multiple bending of the sensor in air and underwater.

As a proof of principle, we fabricated a strain sensor by printing the LM/PASTA composite on a stretchable TPU substrate. LM composite‐based strain sensors working in the dry state were previously reported.^[^ ^29^, ^31^, ^47^ ^]^ To evaluate the suitability of the LM composite for use in aqueous environments, we performed measurements in water after attaching the sensor to the wrist of a volunteer using Tegaderm tape (**Figure** **5**a). The wrist wearing the sensor was repeatedly bent and released in the air and underwater (Figure 5b). The resistance change ($\Delta R/R_{0}$) during wrist bending under dry and wet conditions exhibited a similar behavior (Figure 5c). Additionally, the resistance change in both conditions was close to 2%, indicating that the LM/PASTA‐based strain sensor performs equally well in air and water. This demonstrated the potential of the LM composite as a wearable strain sensor in various underwater applications, including swimming, scuba diving, or rehabilitation in water for monitoring body motions.

### 2.6 Recycling of the Liquid Metal

**Figure 6:**
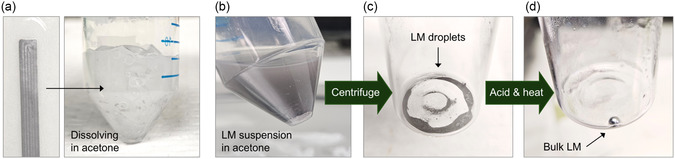
LM recycling process. a) LM/PASTA composite printed on TPU soaked in acetone. b) LM suspension in acetone after removal of the insoluble TPU. c) LM droplets collected after centrifugation. The droplets were separated by native oxide on their surface. d) Bulk LM after adding acetic acid and heating.

Recyclable components and eco‐friendly chemicals are key to sustainable electronics. EGaIn, an alloy of two critical materials, is highly recommended for recycling. Extraction of EGaIn from LM‐based electronics has been achieved using hydrochloric acid and sodium hydroxide.^[^ ^21^, ^24^, ^29^ ^]^ Here, we performed LM recycling using acetone and acetic acid, both of which are classified as green solvents.^[^ ^48^ ^]^ The printed LM composite on TPU was dissolved in acetone (**Figure** **6**a). Since PASTA is soluble in acetone (Table S3, Supporting Information), PASTA‐grafted LM droplets were redispersed in acetone after the removal of the TPU substrate (Figure 6b). The LM collected by centrifugation maintained its droplet state, as the surface oxides prevented the droplets from merging (Figure 6c). After adding acetic acid and applying moderate heat, the droplets merged into reusable bulk LM (Figure 6d). During this step, the following chemical reaction likely occurred on the surface of LM droplets^[^ ^49^ ^]^

(2) $${\text{6CH}}_{3}\text{COOH }\left(l\right)+{\text{Ga}}_{2}O_{3}\;\left(s\right)\to \text{ 2Ga}{\left({\text{CH}}_{3}\text{COO}\right)}_{3}\left(\text{aq}\right)+{\text{3H}}_{2}\text{O }\left(l\right)$$

Upon contact with acetic acid, gallium acetate (Ga(CH_3_COO)_3_) formed on the surface of the LM droplets, replacing the gallium oxide layer. We hypothesize that the gallium acetate on the droplet surface is dissolved by the acetic acid, allowing the pure EGaIn droplets to merge into bulk LM before additional gallium oxide could form. Given that the gallium oxide layer is typically 0.5–5 nm thick,^[^ ^50^ ^]^ the loss of gallium compounds during recycling is negligible. The yield of recycled LM was ≈95%. This demonstrates that the EGaIn in the LM/PASTA composite can be effectively recycled using a simple extraction process with green solvents. Furthermore, compared to other polymer matrices, PASTA facilitates LM recycling, contributing to a sustainable LM composite with high performance (Table S2, Supporting Information).

## 3 Conclusion

In this study, we developed a highly conductive and stretchable printable LM composite with a minimal resistance change under mechanical strain and exceptional stability under cyclic mechanical strain in both dry and wet environments. The composite incorporates a specifically designed block copolymer as the polymer matrix, featuring a polyacrylic acid functional block to interact with LM droplets ensuring suspension stability, and a poly(styrene‐*ran*‐butyl acrylate) block to provide softness, stretchability, and hydrophobicity. The resulting material met the key requirements for a stretchable conductor: metallic conductivity (in the 10^5^ S m^−1^ range), high stretchability (≈500%), and minimal resistance change upon strain (≈5% at 30% strain). The LM composite exhibited exceptional durability with negligible degradation in electrical performance for 16 h of cyclic strain at 50% in both dry and wet conditions, which made possible its use in underwater strain sensors. In addition to its impressive performance, the composite can be easily dissolved in green organic solvents to recover valuable LM, promoting the recycling of critical materials.

## 4 Experimental Section

4.1

4.1.1

##### Materials

Azobisisobutyronitrile (AIBN, Millipore Sigma) was purified by recrystallization from ethanol and dried in a vacuum oven at 50 °C for ≈16 h. EGaIn (Ga 75.5%/In 24.5%, ≥99.99%), 2‐(dodecylthiocarbonothioylthiododecylthiocarbonothioylthio)‐2‐methylpropionic acid (DDMAT) (98%), acrylic acid (99%), n‐hexane (≥95%), diethyl ether (≥99%), tetrahydrofuran (THF) (≥99.5%), cyclohexane (≥99%), and 1‐Octanol (≥99%) were purchased from Millipore Sigma and used as received. *N*,*N*‐dimethylformamide (DMF) (99.8%), butyl acrylate (>99%), styrene (99%), toluene (99.8%), acetic acid (99.7%), acetone (99.5%), and anisole (99%) were purchased from Thermo Fisher Scientific. Thermoplastic polyurethane (TPU) on removable silicone paper (Elecrom Stretch White, thickness 80 μm) was purchased from Policrom Screens (Italy).

##### Polymer Synthesis

The synthesis of the polymer PASTA consisted of two steps: 1) synthesis of DDMAT‐terminated poly (acrylic acid) (PAA) via RAFT polymerization (Figure 1a) and 2) free radical random polymerization of PASTA using the synthesized DDMAT‐terminated PAA and monomers (styrene and butyl acrylate) (Figure 1b). Both RAFT and random polymerization were conducted following previously reported procedures.^[^ ^51^, ^52^ ^]^ The ratios of reagents in all reactions were determined based on the reaction stoichiometry. Through preliminary tests of different monomer ratios, we selected the one in which the polymer exhibited rubber‐like mechanical properties, assessed by manual stretching.

To synthesize DDMAT‐terminated PAA, AIBN (23 mg, 0.14 mmol), as the initiator, and DDMAT (50 mg, 0.14 mmol), as the chain transfer agent, was introduced into a round‐bottom flask, which was sealed with a rubber septum and purged with N_2_ gas for 10 min. DMF (3.63 g, 3.85 mL) was added, and the mixture was stirred for 5 min at room temperature. Acrylic acid (1.48 g, 0.02 mol) was successively added to the mixture, and then polymerization was conducted at 80 °C for 2 h while stirring with a magnetic stirrer. The reaction was stopped by cooling the flask to room temperature in a water bath. The resulting DDMAT‐terminated PAA dissolved in DMF was purified by precipitation in hexane/diethyl ether (1:1 *w* *w* ^−1^) twice, and the precipitate was collected with a filter paper (Whatman quantitative filter paper, 16 μm pore size) using a vacuum pump. The solid DDMAT‐terminated PAA was then dried in a vacuum oven (VWR 1400 E) at 50 °C for ≈16 h. This process resulted in a reaction yield of ≈90%, which was determined by comparing the weight differences between the synthesized polymer and monomers used in the reaction.

To synthesize PASTA, AIBN (45 mg, 0.27 mmol) and 50 mg of the synthesized DDMAT‐terminated PAA were introduced into a round‐bottom flask, which was sealed with a rubber septum and purged with N_2_ gas for 10 min, then the DMF (21.30 g, 22.56 mL) solvent was added, and the temperature was increased to 80 °C. Butyl acrylate (10.46 g, 0.82 mol) and styrene (3.64 g, 0.35 mol) were added, and polymerization was carried out at 80 °C for 24 h. PASTA dissolved in DMF was precipitated twice in hexane/diethyl ether (10:1, *w* *w* ^−1^). The precipitate was filtered through filter paper using a vacuum pump. The solid polymer was dried in a vacuum oven at 50 °C for ≈16 h (yield of ≈85%).

##### Polymer Characterization

The synthesized DDMAT‐terminated PAA and PASTA samples were examined in deuterated chloroform using ^1^H‐NMR spectroscopy (Figure S1, Supporting Information). Spectra were obtained using a Bruker AVANCE II 400 spectrometer operating at 400 MHz to confirm the chemical structures of DDMAT‐terminated PAA and PASTA. For GPC sample preparation to determine polymer molecular weight distribution, PASTA was diluted to a concentration of 0.2 wt% in THF. The analysis was performed using a 1260 Infinity GPC system equipped with a WAT044228 Styragel HR 5 E column manufactured by Waters. THF was used as the eluent and was maintained at 30 °C at a flow rate of 1 mL min^−1^. In this study, we achieved a polydispersity index (PDI) of 2 for free‐radical polymerization (Figure S2, Supporting Information). DSC analysis (Q2000, TA Instruments, USA) was conducted to determine the glass transition temperature (*T*_g_) of the PASTA samples. ≈12 mg of the sample were loaded into a standard aluminum pan and subjected to a constant flow of nitrogen. To ensure the accurate representation of the thermal behavior without thermal history, the samples were initially cooled to −30 °C and then heated to 200 °C at a rate of 10 °C min^−1^. Subsequently, a second heating cycle was performed from −30 to 200 °C at the same heating rate. The analysis was based on data collected during the second heating cycle. To prepare the samples for tensile tests, 50 wt% PASTA was dissolved in DMF. The solution was drop cast in a Teflon dog‐bone mold and dried at 30 °C on a hotplate for 24 h. PASTA dog bone with ≈4.1 mm width, ≈10.3 mm length, and ≈1.2 mm thickness was obtained. The tensile tests were performed using a Mach‐1 V500cst MA009 mechanical tester (Biomomentum Inc., Canada) at a stretching speed of 500 mm min^−1^.

##### Preparation of LM/PASTA Ink

To study the effects of the polymer content on the composite properties, the LM/PASTA suspensions were prepared by dissolving various amounts of PASTA (15, 35, and 70 mg) in toluene (3 mL) in a 20 mL glass vial and adding EGaIn (1.1 g). These polymer‐to‐LM ratios were selected in agreement with previous studies of polymer‐grafted LM droplets. The mixture was placed in a cooling water bath at room temperature and subjected to tip sonication (450 Sonifier, Branson, 400 W) for 12 min to obtain uniform suspensions. The LM composites containing 15, 35, and 70 mg PASTA were named P15, P35, and P70, respectively. The suspensions were then stored under ambient conditions in sealed glass vials.

##### Suspension Stability Test

To demonstrate the effect of PASTA as a surface ligand, an LM suspension was prepared with EGaIn (2.2 g) and PASTA (70 mg) in toluene (6 mL) by tip sonication, as previously described. A control LM suspension was prepared using EGaIn (2.2 g) and pure toluene (6 mL). The resulting LM suspensions were observed for 7 days.

PASTA was found to be soluble in DMF, toluene, anisole, acetone, *o*‐xylene, and tetrahydrofuran (Table S3, Supporting Information). However, the composite in DMF flocculated within an hour (Figure S16, Supporting Information). Polar solvents with high dipole moments, such as DMF, may interfere with the interaction between the oxide layer of the droplets and the carboxyl groups of the polymer ligands,^[^ ^42^ ^]^ leading to poor suspension stability. Among the other nonpolar solvents tested, toluene was selected for the preparation of the LM suspension because of its relatively low toxicity and chemical compatibility with our printing equipment.

##### Printing of LM/PASTA Ink

The LM/PASTA composite was printed on thermoplastic polyurethane (TPU) substrates using a direct ink writing printer (Voltera NOVA) with a 30‐gauge stainless steel dispensing nozzle (Nordson EFD) attached to a 3 cc syringe. The patterns were designed using the DipTrace software. The printed materials were dried at room temperature because of the high volatility of toluene. The dried samples were treated with a 10 vol% acetic acid solution in acetone by drop casting with a micropipette and then dried at 80 °C for 30 min. The samples were stored under ambient conditions prior to characterization. The TPU substrates with the printed composites were peeled off from the removable silicone paper lining before characterization.

##### Scanning Electron Microscopy (SEM)

The morphologies of the printed materials were studied using a Quattro Environmental SEM (Thermo Fisher Scientific) at 5 kV. The elemental composition and mapping were obtained using EDX at 5 kV.

##### Electrical and Electromechanical Characterization

The samples (length = 25 mm, width = 2 mm) were treated with acetic acid solution and mechanically activated with 50% strain 20 times.

The electrical resistance was measured using a four‐point probe (Jandel) connected to a source‐measure unit (SMU, Agilent A1902). The thickness (*d*) of the composite was measured using a dynamic white‐light interferometer (Fogale Nanotech Photomap 3‐D). The conductivities (*σ*) of the LM composites were calculated as follows.

(3) $$\sigma =\frac{1}{R_{s}d}$$

where *R*_s_ denotes the sheet resistance of the sample.

The electromechanical characterization was performed using a uniaxial homemade translational manipulator equipped with (Agilent A1902). The sample underwent varying strains while being subjected to a constant voltage of 30 mV, and the current was recorded to monitor electrical stability. To test the electromechanical properties, the LM composite was stretched to 500% strain at a speed of 2 mm s^−1^. The electromechanical durability test was performed with 5000 cycles of 50% repeated tensile strain at a speed of 5 mm s^−1^. To test the durability of the composite in the wet state, water was continuously delivered to the composite surface using a syringe pump at a flow rate of 0.2 mL h^−1^. The SEM images of two samples used in the dry state durability test were obtained while one was stretched at 50% strain, and another remained without strain.

##### Strain Sensor

Strain sensors (length = 20 cm, width = 2 mm) were printed on the TPU substrates. The sensors were activated by acid treatment followed by stretching to 50% strain 20 times. For on‐body measurements, the sensor was attached to the wrist of a volunteer using Tegaderm tape, and an electrical connection to the SMU was established using copper tape with alligator clamps. The change in current with wrist bending was recorded under a constant applied voltage of 0.03 V. To validate the sensor performance underwater, the same measurement was conducted while the volunteer's hand was immersed in water in a 4 L beaker. Informed written consent was obtained from the volunteer for the test. The protocol for human experiments was approved by the ethical committee of Polytechnique Montréal (approval number CER‐ 2021‐04‐D).

##### Recycling of EGaIn

For recyclability tests, the printed LM composite on a TPU substrate was soaked in acetone for 30 min. This process dissolved the LM composite, and undissolved TPU was manually removed from the solution using tweezers. The LM suspension in acetone was centrifuged at 1500 rpm for 15 min to separate the polymer (supernatant) from LM droplets. The supernatant was discarded, and the remaining LM droplets were transferred to a 20 mL glass vial. Finally, concentrated acetic acid (1 mL, 99.7%) was added to the vial, which was heated at 100 °C on a hot plate until the acid was fully evaporated, leaving bulk EGaIn. The recycling yield was calculated by comparing the weights of EGaIn in the printed composite and that of recycled bulk EGaIn.

##### Statistical Analysis

All data mentioning sample size (*n*) were represented by their mean standard deviation.

## Conflict of Interest

The authors declare no conflict of interest.

## Author Contributions

**Chi‐hyeong Kim**: conceptualization (lead); formal analysis (lead); investigation (lead); methodology (lead); validation (lead); writing—original draft (lead); writing—review and editing (supporting). **Jinsil Kim**: formal analysis (equal); investigation (equal); methodology (equal); writing—review and editing (supporting). **Jiaxin Fan**: formal analysis (equal); writing—review and editing (equal). **Meijing Wang**: investigation (supporting); writing—review and editing (supporting). **Fabio Cicoira**: conceptualization (lead); data curation (equal); formal analysis (equal); funding acquisition (lead); investigation (equal); methodology (lead); project administration (lead); resources (lead); software (supporting); supervision (lead); validation (equal); visualization (equal); writing—original draft (supporting); writing—review and editing (lead).

## Supporting information

Supplementary Material

## Data Availability

The data that support the findings of this study are available from the corresponding author upon reasonable request.
