# Supplementary material for: Recyclable Printed Liquid Metal Composite for Underwater Stretchable Electronics
Source: Small Sci. 2025 Mar 31;5(5):2400553. doi: 10.1002/smsc.202400553 (PMC12087783; doi:10.1002/smsc.202400553)
Supplement: Supplementary file 1 — Supplementary Material [file SMSC-5-2400553-s001.pdf]

Supporting Information

**Recyclable Liquid Metal Composite for Underwater Stretchable Electronics**

*Chi-hyeong Kim, Jinsil Kim, Jiaxin Fan, Meijing Wang, and Fabio Cicoira**

Department of Chemical Engineering, Polytechnique Montreal, Canada, H3T 1J4
E-mail: fabio.cicoira@polymtl.ca

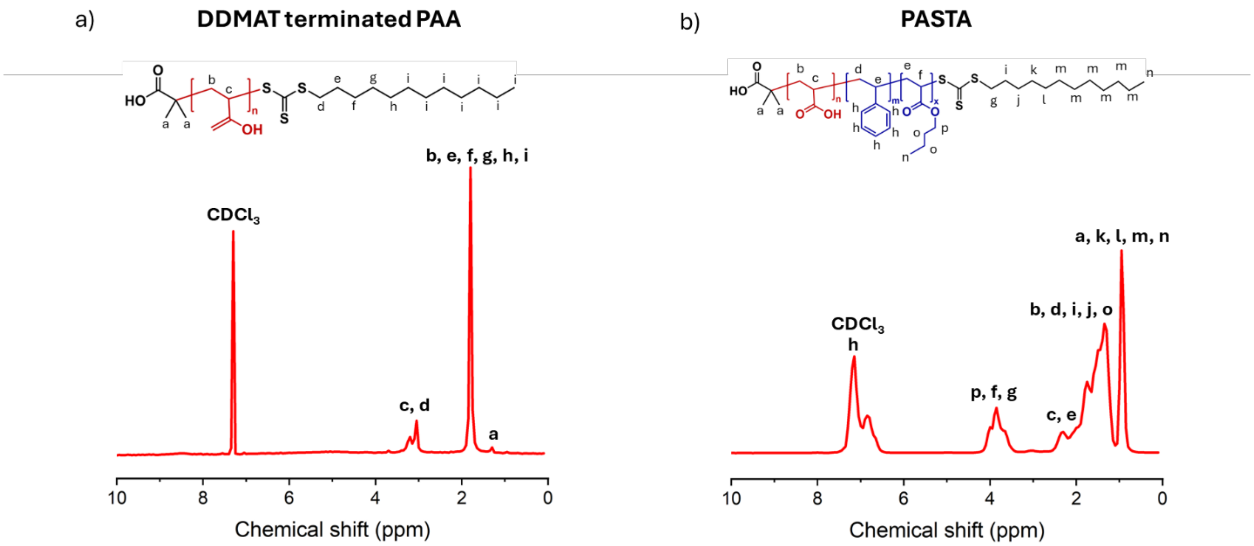


**Figure S1.** $^{1}$H-NMR of a) DDMAT-terminated PAA and b) PASTA. The chemical structures of the synthesized polymers were verified with $^{1}$H-NMR.

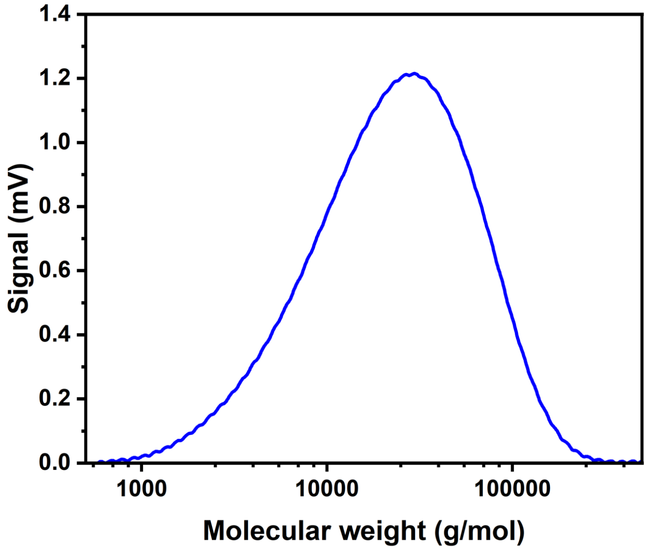


| | Mw (kg/mol) | Mn (kg/mol) | PDI |
|---|---|---|---|
| PASTA | 68 ± 1 | 34 ± 1 | 2 ± 0.1 |

**Figure S2.** GPC of PASTA. The molecular weight averages and PDI of PASTA were extracted from the GPC data.

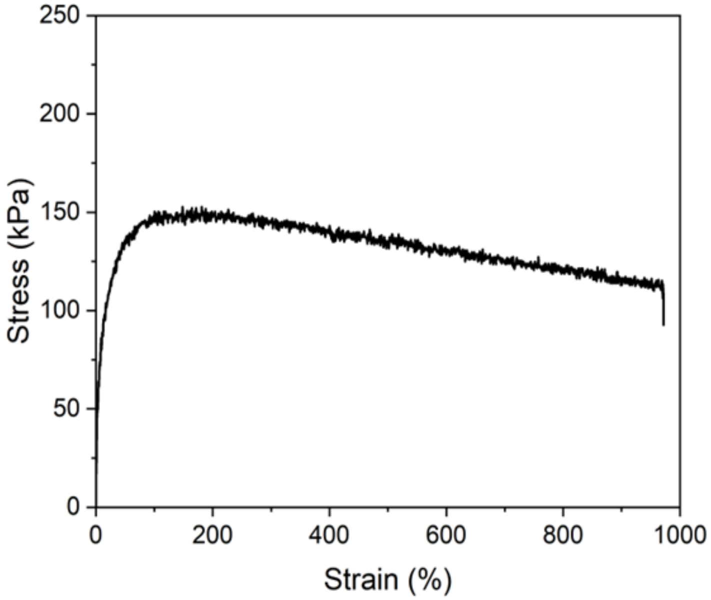


**Figure S3.** Tensile stress-strain curve of PASTA.

Mechanical characterization achieved via tensile stress-strain curves indicates that free-standing polymer films exhibit an elastic deformation up to 40% strain and are stretchable up to at least 900%. Additionally, for applied strains higher than 100%, we observed a decrease in the stress as the strain increased. The behavior can be correlated with the low glass transition temperature ($T_g$) of the material since the linear chains of PASTA may have high mobility at temperatures higher than $T_g$ and can show a stress decrease in the mechanical ductile region.

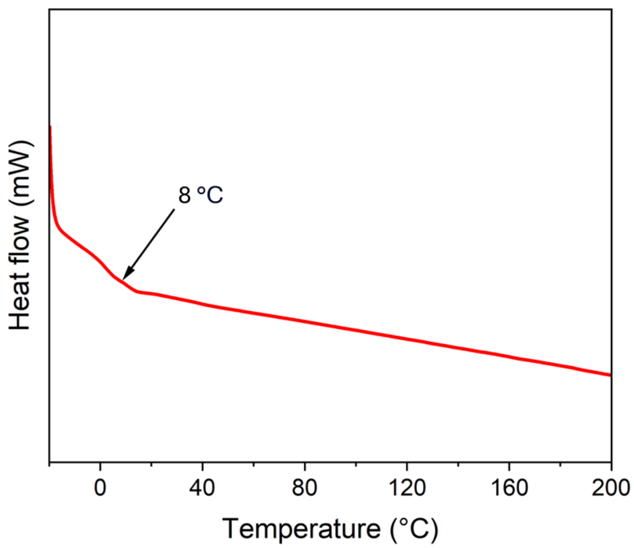


**Figure S4.** Differential scanning calorimetry of PASTA.

From DSC, $T_g$ of PASTA is around 8°C. Hence, PASTA demonstrated high stretchability and low $T_g$, which are suitable properties for LM composite development.

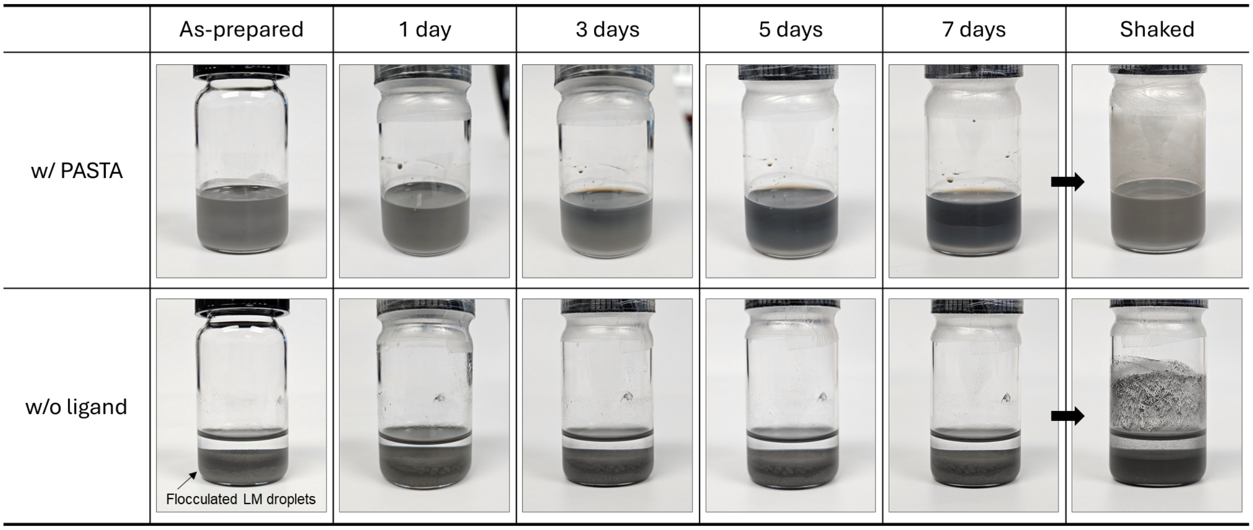


**Figure S5.** Digital Photos of LM suspension stability test over time.

The LM/PASTA suspension was gradually less homogeneous starting from day 3 after preparation, indicating the LM droplets slowly flocculated due to gravity. A clear separation layer in the LM suspension was observed on day 7. However, the polymer-grafted LM suspension could recover its homogeneity after shaking by hand, as reported in the literature,[1] whereas sedimented LM droplets were observed after shaking for the LM suspension prepared without the polymer ligand.

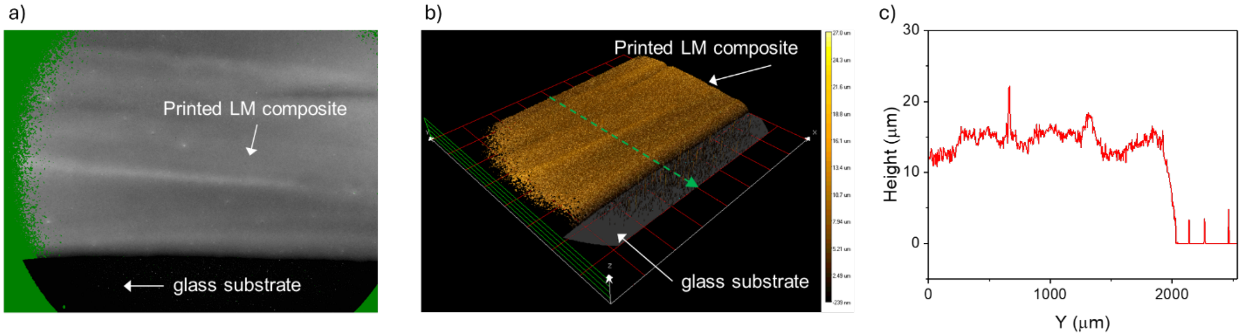


**Figure S6.** Surface profile of a printed LM composite measured by the white light interferometer. a) Optical and b) 3D images of the composite. c) Cross-sectional profile of the composite, which was measured at the green dash line in the 3D image in b). The average thickness is 14.00 ± 3.00 μm ($n$ = 5).

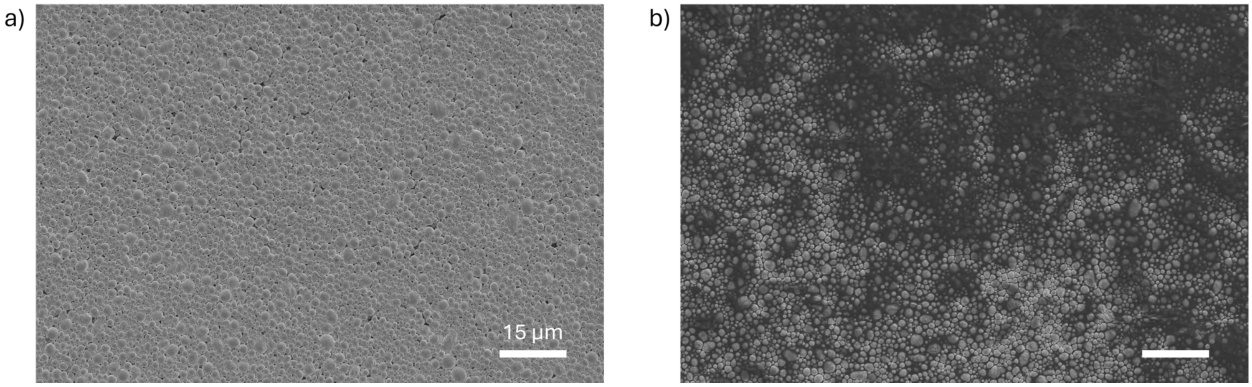


**Figure S7.** SEM images of a) as-printed P35 composite and b) acid-treated P35 composite.

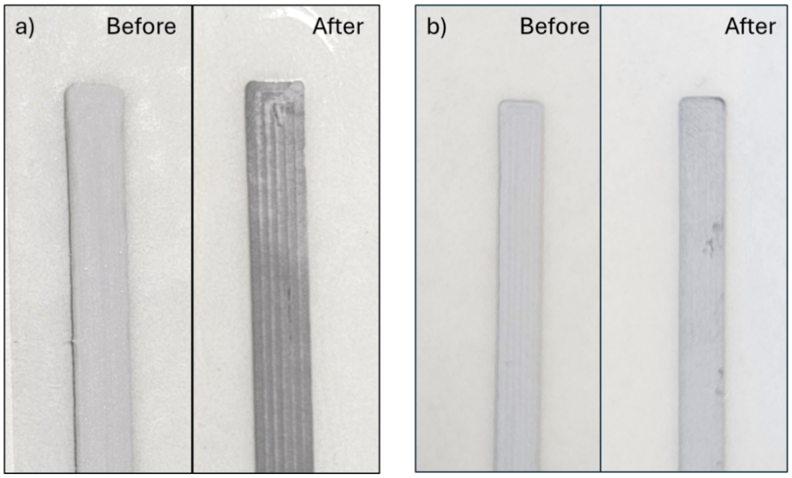


**Figure S8.** Optical images of as-printed P35 before & after a) acid solution and b) acetone treatment. All the printed LM composites have a 2 mm width.

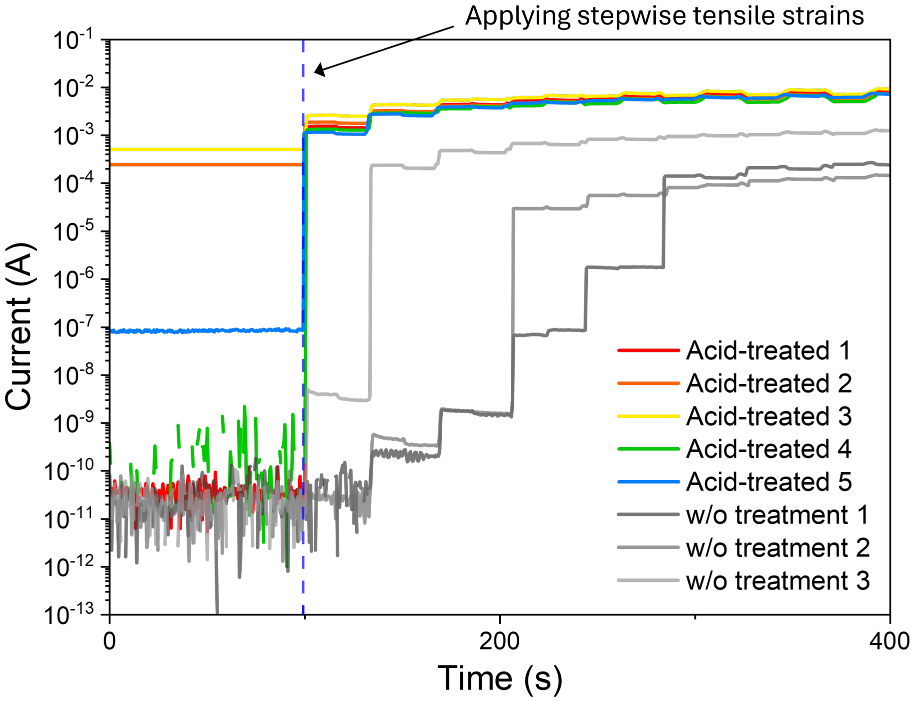


**Figure S9.** Currents vs time plot in log scale, comparing combined chemical and mechanical activation (*n*=5) with mechanical activation alone (*n*=3) from 0 to 400 seconds in Figure 3c. The initial currents, before mechanical activation, are at the noise level ($10^{-11}$-$10^{-10}$ A) for most samples. A few acid-treated composites (Acid-treated 2,3, and 5) showed higher current ($10^{-7}$-$10^{-3}$ A) before activation, likely due to accidental activation while handling. However, the minor activation did not affect the electrical properties of fully activated LM composites.

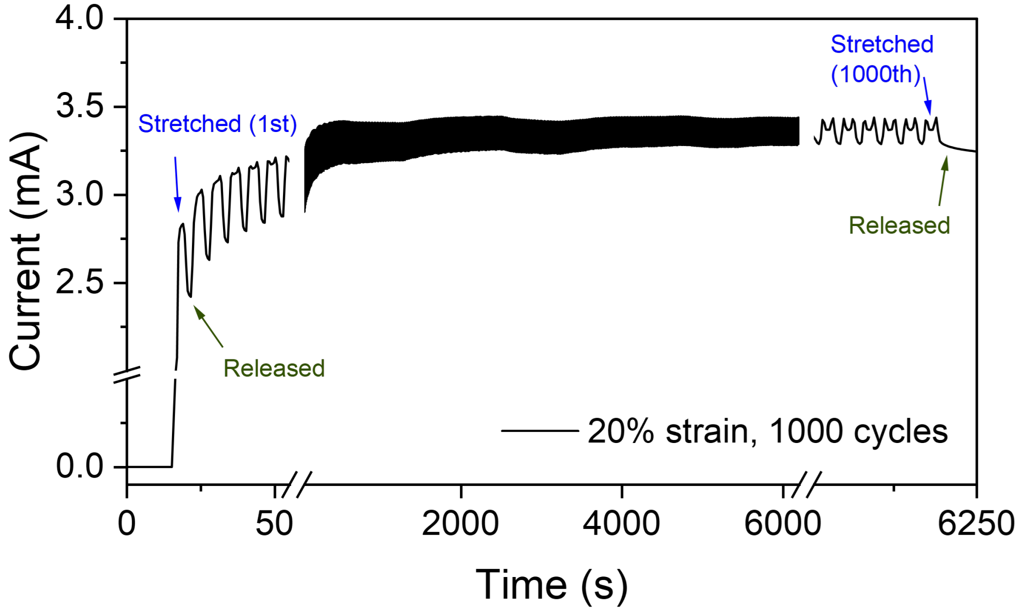


**Figure S10.** The current of P35 while applying 20% strain 1000 times. The current in 0-55 and 6200-6250 second were enlarged.

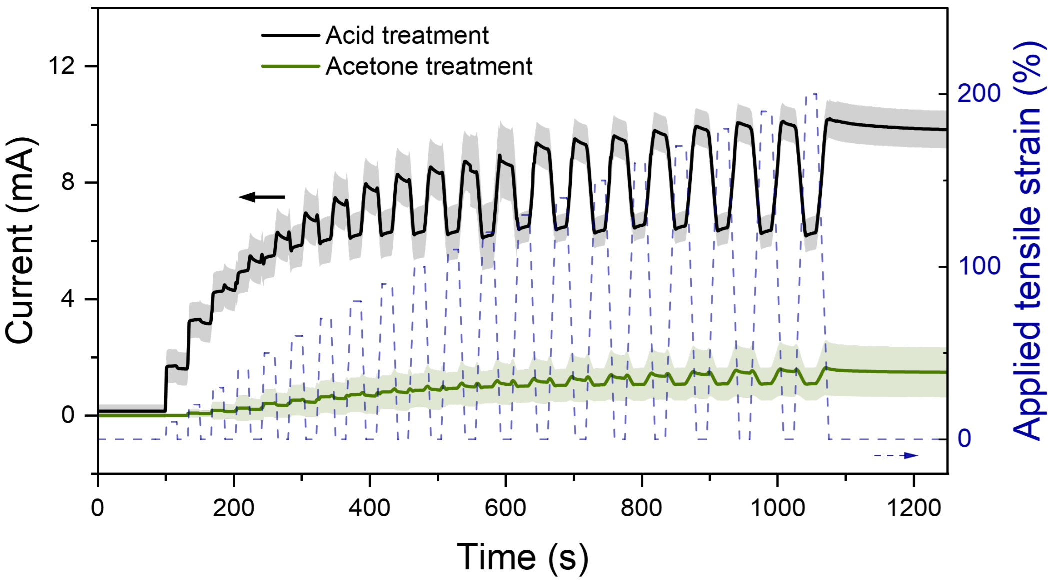


**Figure S11.** Comparison of the currents of acid-treated (*n*=5) and acetone-treated (*n*=3) LM composites during mechanical activation.

**Table S1.** Electrical conductivities of LM composites (*n*=5). P15, P35, and P70 were prepared with 15 mg, 35 mg, and 70 mg PASTA, respectively. The composites were mechanically activated with 50% tensile strain 20 times before the measurement.

| | Chemical Treatment | Conductivity [S/cm] |
|---|---|---|
| P15 | Acid | 3555 ± 875 |
| P35 | Acid | 2770 ± 286 |
| P35 | Acetone | 288 ± 136 |
| P35 | x | 196 ± 86 |
| P70 | Acid | 429 ± 108 |

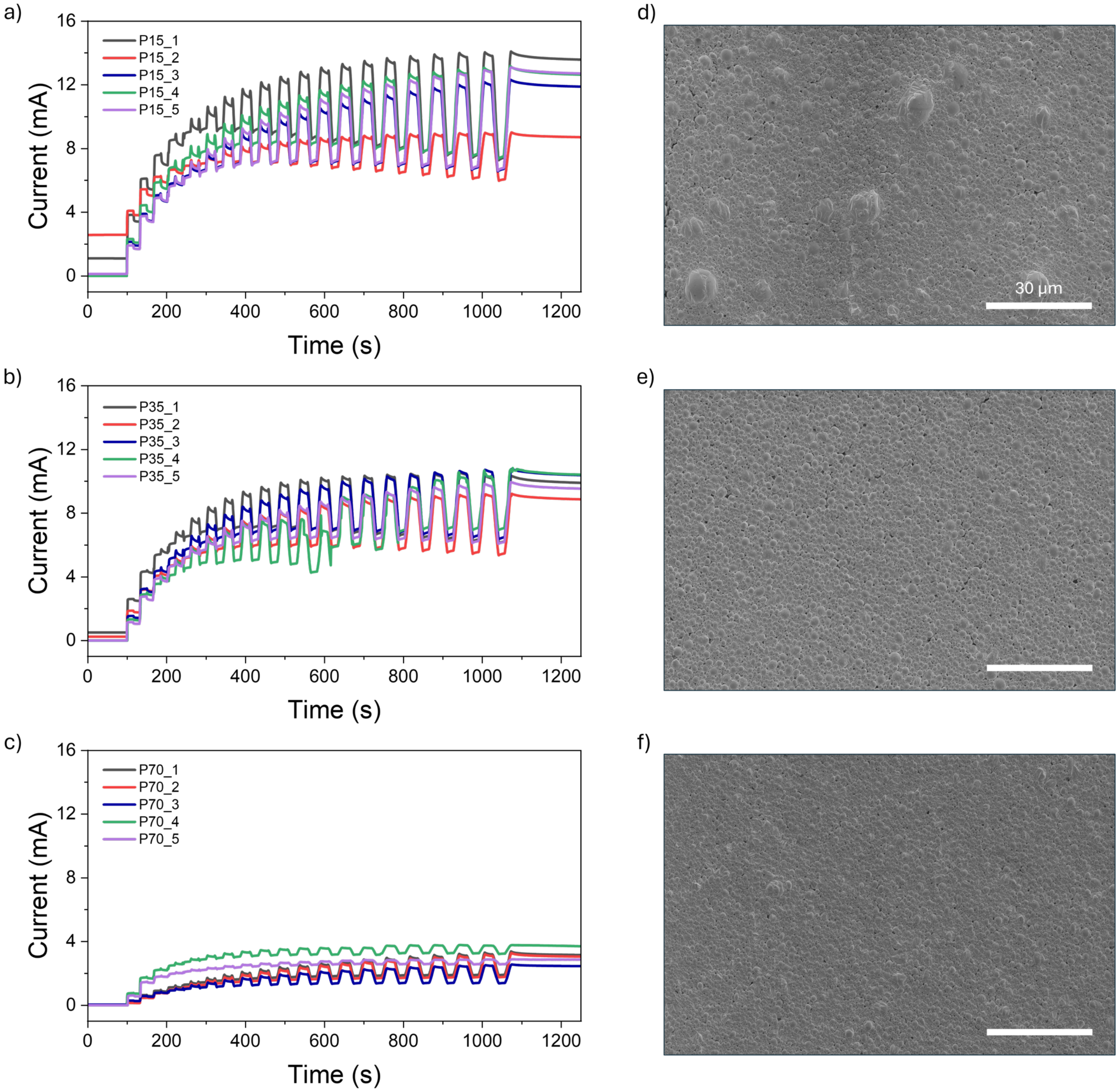


**Figure S12.** The mechanical activation test of acid-treated LM/PASTA containing a) 15 mg, b) 35 mg, and c) 70 mg polymer ($n$ = 5). The applied tensile strain is increased by a 10% increment from 0% to 200%. SEM images of as-printed d) P15, e) P35, and f) P70.

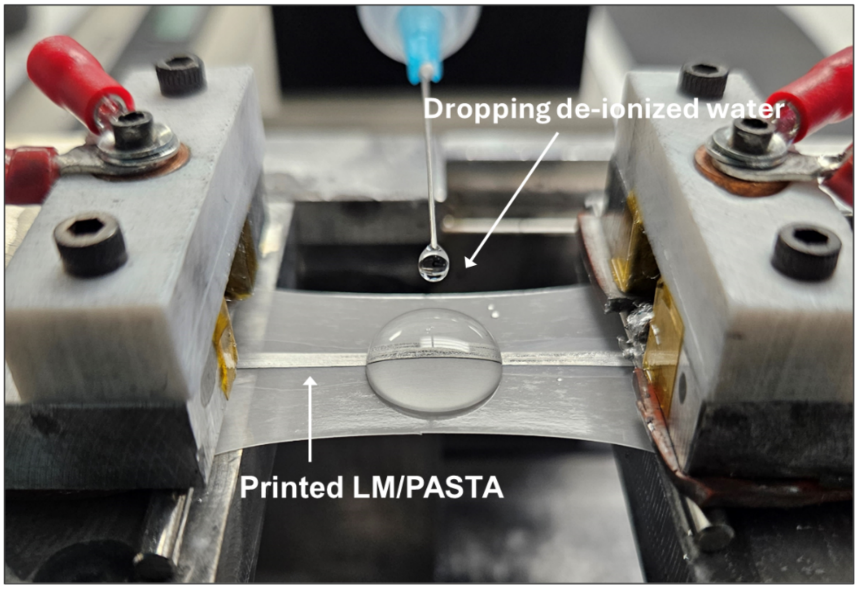


**Figure S13.** Digital photograph of the electromechanical durability test setup with a printed LM composite under the wet condition.

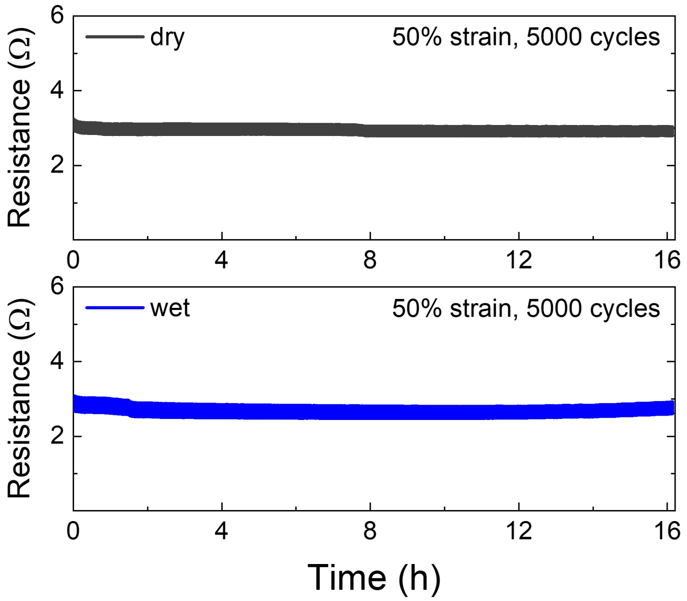


**Figure S14.** Resistance vs. time of LM/PASTA (P35) over 5000 cycles of 50% strain conducted over 16 h under dry and wet conditions. The samples were prepared with the exact same conditions as the composites used in Figure 4e in the main text.

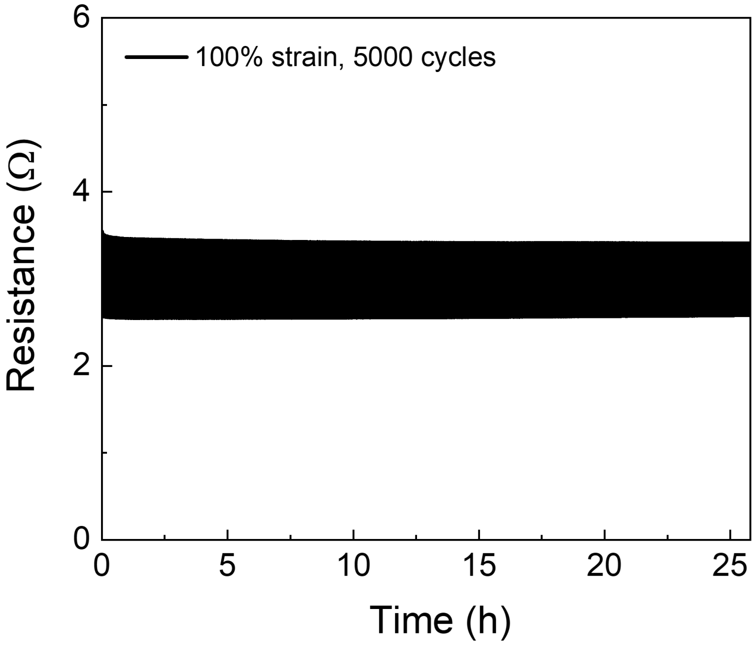


**Figure S15.** Resistance vs cycle numbers of the dry LM/PASTA (P35). The tests were conducted with 100% tensile strain for 5000 cycles at a speed of 5 mm $s^{-1}$.

**Table S2.** Comparison of LM composite-based stretchable conductors

| LM | Polymer | Conductivity [$10^5$ S $m^{-1}$] | Stretchability[a)] [%] | Stability in water | Recyclability | Ref |
|---|---|---|---|---|---|---|
| EGaIn | PASTA | 0.4-3.6 | 500 | Yes | Yes | **This work** |
| EGaIn | Poly(styrene-isoprene-styrene) | N/A | 500 | N/A | Yes | [2] |
| EGaIn | Poly(styrene-isoprene-styrene) and polybutadiene | 0.2 | 1200 | N/A | Yes | [3] |
| EGaIn | Polyester polyol-rich TPU | 22.5 | 2200 | N/A | Yes | [4] |
| EGaIn | PVA | 1.3 | 20 | N/A | Yes | [5] |
| EGaIn | PSS | 15.0 | 500 | N/A | Yes | [6] |
| EGaIn | PDMS | 1.4 | 50 | N/A | N/A | [7] |
| Galinstan | PDMS | 1.2 | 75 | N/A | N/A | [8] |
| EGaIn | PDMS | N/A | 128 | N/A | N/A | [9] |
| EGaIn | PDMS and Ecoflex | N/A | 40 | Yes[b)] | N/A | [10] |

[a)] The maximum stretchability exhibited in each work was surveyed.

[b)] Compared to this work, which showed that LM/PASTA as a stretchable conductor is electromechanically durable in water, they demonstrated the electrical stability of their unstrained composites in water.

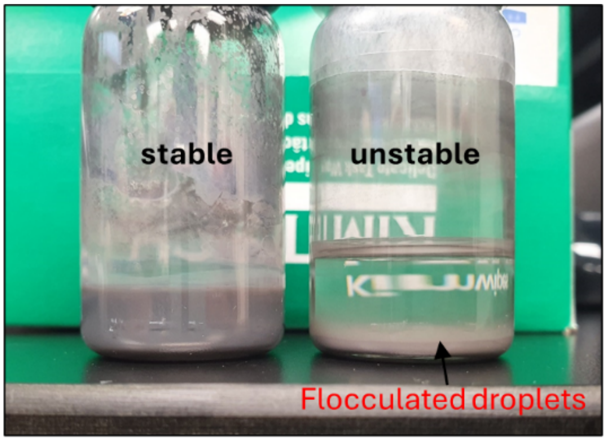


**Figure S16.** Digital photograph of LM suspensions. The vial on the left contains suspension in toluene, which has well-dispersed LM droplets after a day. The suspension on the right containing DMF solvent has flocculated droplets on the bottom one day after preparation.

The effects of polar solvents on the stability of suspension were shown here. PASTA-grafted LM suspension with toluene on the left side is well-dispersed a day after preparation. On the other hand, the LM droplets in the suspension prepared with PASTA dissolved in DMF solvent flocculated, indicating it is not suitable for ink preparation.

**Table S3** Solubility test of polymer and LM suspension stability test in various solvents

| Solvent | Solubility of the polymer | Suspension stability | Dipole moment |
|---|---|---|---|
| Toluene | O | O | 0.36 D |
| *orth*-Xylene | O | O | 0.45 D |
| Anisole | O | O | 1.38 D |
| THF | O | O | 1.75 D |
| DMF | O | X | 3.82 D |
| Acetone | O | N/A | 2.88 D |
| Cyclohexane | X | N/A | 0 D |
| 1-Octanol | X | N/A | 1.68 D |
| DMSO | X | N/A | 3.96 D |
| Water | X | N/A | 1.85 D |

Through solvent tests, we found that PASTA was soluble in DMF, toluene, anisole, xylene, THF, and acetone. Solvent polarity is correlated to the dipole moment of the polymer, indicating DMF and DMSO are polar solvents. It was reported that the polar solvent DMSO can detach the polymer grafted on LM droplets.[11] We believe that DMF may have the same effect, leading to flocculation for a short time. Toluene was selected as the solvent for LM composite ink development since it is compatible with the printer parts and relatively less toxic than the other nonpolar solvents.

**Supporting Note**

Calculation of the theoretical resistance for a sample with a constant conductivity[4]

The traditional equation of resistance $R(t)$ at a given time $t$ can be expressed as follows:

$$R(t) = \rho(t) \times \frac{L(t)}{A(t)} = \frac{1}{\sigma(t)} \times \frac{L(t)^2}{V(t)} \quad \text{(S1)}$$

where $\rho(t)$, $\sigma(t)$, $L(t), A(t)$,and $V(t)$ are resistivity, conductivity, length, cross-sectional area, and volume of the material at a given time $t$. When t=0, the resistance and the conductivity of unstretched material are $R_0$ and $\sigma_0$, and the dimension factors are $L_0$, $A_0$, and $V_0$. Hence, the resistance under strain with respect to the original value can be expressed as:

$$\frac{R(t)}{R_0} = \frac{\sigma_0 \cdot V_0}{\sigma(t) \cdot V(t)} \times \frac{L(t)^2}{{L_0}^2} \quad \text{(S2)}$$

When assuming the conductivity and the volume remain constant while stretching the material, Equation (S2) can be simplified as:

$$\frac{R(t)}{R_0} = \left(1 + \varepsilon(t)\right)^2 \quad \text{(S3)}$$

where $\varepsilon(t)$ is the strain corresponding to $(L(t)/L_0)$.

## Reference


[1] Q. Wei, M. Sun, Z. Wang, J. Yan, R. Yuan, T. Liu, C. Majidi, K. Matyjaszewski, *ACS Nano* **2020,** *14*, 9884-9893.

[2] A. Hajalilou, E. Parvini, J. P. M. Pereira, P. A. Lopes, A. F. Silva, A. De Almeida, M. Tavakoli, *Adv. Mater. Technol.* **2023,** *8*, 2201621.

[3] R. Tutika, A. B. M. T. Haque, M. D. Bartlett, *Commun. Mater.* **2021,** *2*, 64.

[4] S. Chen, S. Fan, J. Qi, Z. Xiong, Z. Qiao, Z. Wu, J. C. Yeo, C. T. Lim, *Adv. Mater.* **2023,** *35*, 2208569.

[5] J. Xu, H. Guo, H. Ding, Q. Wang, Z. Tang, Z. Li, G. Sun, *ACS Appl. Mater. Interfaces* **2021,** *13*, 7443-7452.

[6] G. H. Lee, Y. R. Lee, H. Kim, D. A. Kwon, H. Kim, C. Yang, S. Q. Choi, S. Park, J. W. Jeong, S. Park, *Nat. Commun.* **2022,** *13*, 2643.

[7] E. J. Markvicka, M. D. Bartlett, X. Huang, C. Majidi, *Nat. Mater.* **2018,** *17*, 618-624.

[8] G. Wang, J. Chen, W. Zheng, B. Shen, *Chem. Eng. J.* **2024,** *488*, 151052.

[9] K. Schlingman, G. M. D'Amaral, R. S. Carmichael, T. B. Carmichael, *Adv. Mater. Technol.* **2022,** *8*, 2200374.

[10] E. J. Barron, E. T. Williams, B. T. Wilcox, D. H. Ho, M. D. Bartlett, *J. Polym. Sci.* **2023,** *62*, 3818-3829.

[11] G.-H. Lee, H. Kim, J. Lee, J.-Y. Bae, C. Yang, H. Kim, H. Kang, S. Q. Choi, S. Park, S.-K. Kang, J. Kang, Z. Bao, J.-W. Jeong, S. Park, *Mater. Today* **2023,** *67*, 84-94.
